# Supplementary material for: A short intrinsically disordered region at KtrB’s N-terminus facilitates allosteric regulation of K+ channel KtrAB
Source: Nat Commun. 2025 May 7;16:4252. doi: 10.1038/s41467-025-59546-z (PMC12059179; doi:10.1038/s41467-025-59546-z)
Supplement: Supplementary file 1 — Supplementary Information [file 41467_2025_59546_MOESM1_ESM.pdf]

Supplementary data

A short intrinsically disordered region at KtrB's N-terminus facilitates allosteric regulation of K^+ channel KtrAB

Janina Stautz[1,#], David Griwatz[1,#], Susann Kaltwasser[2], Ahmad Reza Mehdipour[3,4], Sophie Ketter[1], Celina Thiel[1], Dorith Wunnicke[1], Marina Schrecker[1], Deryck J. Mills[5,+], Gerhard Hummer[4,6], Janet Vonck[5,*], Inga Hänelt[1,*]

[1] Institute of Biochemistry, Goethe University Frankfurt, Frankfurt am Main, Germany.

[2] Central Electron Microscopy Facility, Max Planck Institute of Biophysics, Frankfurt am Main, Germany

[3] Center for Molecular Modeling, Ghent University, Zwijnaarde, Belgium

[4] Department of Theoretical Biophysics, Max Planck Institute of Biophysics, Frankfurt am Main, Germany

[5] Department of Structural Biology, Max Planck Institute of Biophysics, Frankfurt am Main, Germany

[6] Institute for Biophysics, Goethe University Frankfurt, 60438 Frankfurt am Main, Germany

[#] These authors contributed equally.

[+] deceased

* These authors jointly supervised this work.

Email: janet.vonck@biophys.mpg.de (JV)

Email: haenelt@biochem.uni-frankfurt.de (IH)

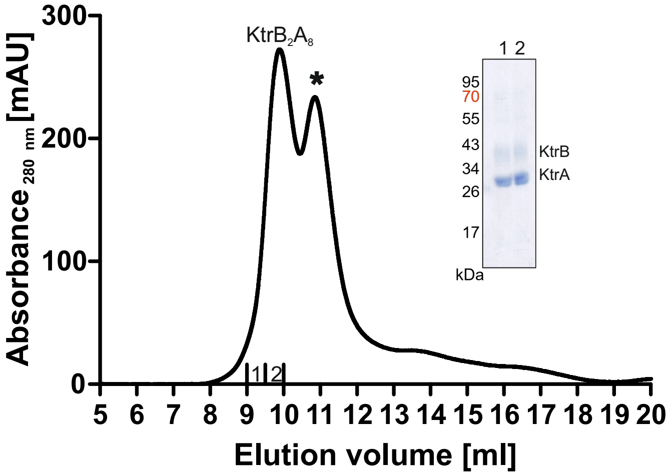

Supplementary Fig. 1: Size-exclusion chromatography of purified $KtrB_2A_8$. Elution profile of the intact assembled KtrAB complex performed on a Superdex 200 Increase 10/300 GL column. In addition to KtrAB, free KtrA rings co-elute at higher elution volumes (indicated by *), as KtrA is supplied in great excess during purification. Sample composition of KtrAB peak fractions (labelled 1 and 2, respectively) was analysed on a Coomassie-stained 12% SDS PAGE (inset) and the presence of KtrA (28 kDa) and KtrB (50kDa, migrates at 37 kDa) was confirmed.

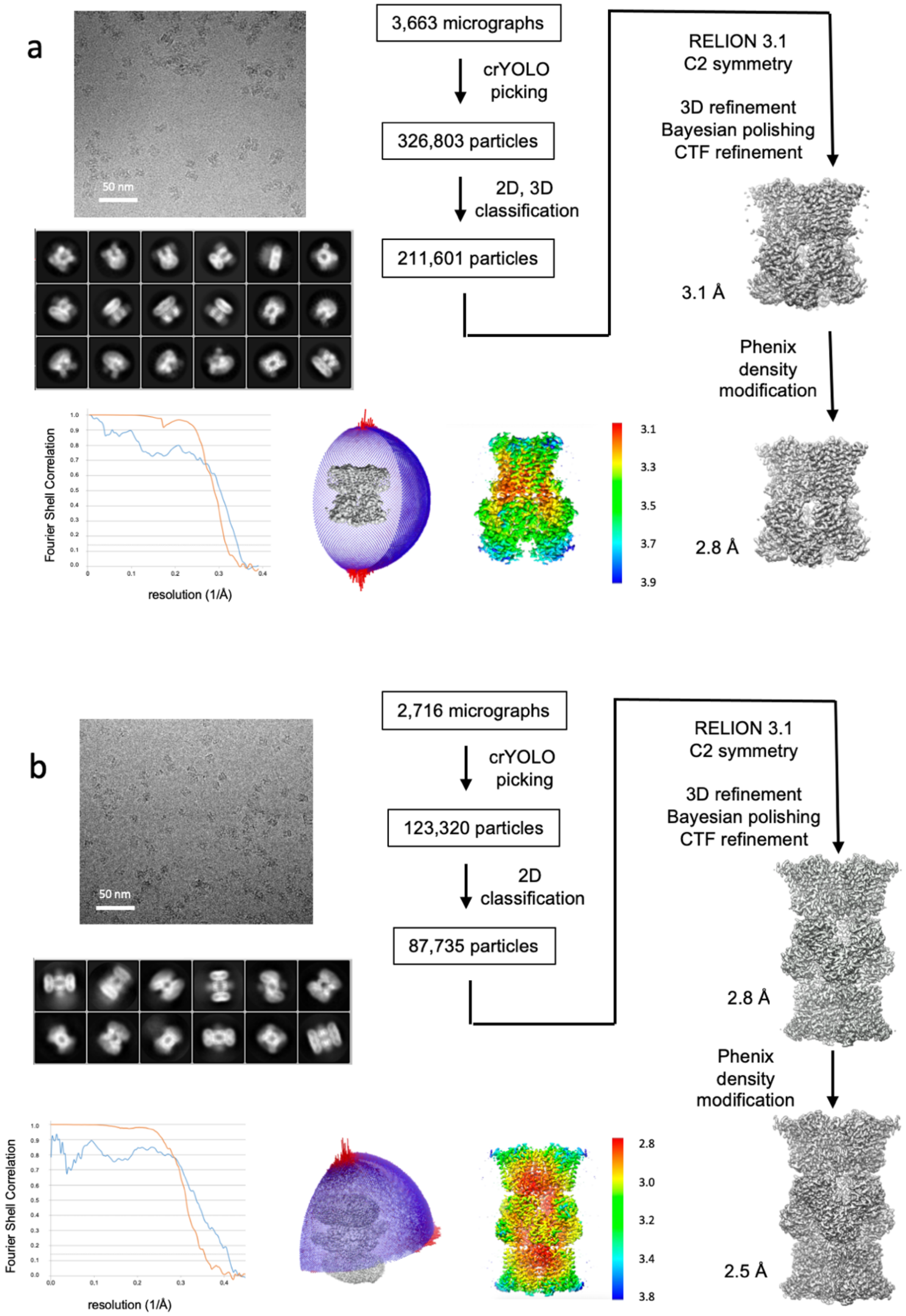

Supplementary Fig. 2: Cryo-EM workflow. a Native KtrAB complex: representative micrograph, selected 2D classes, processing workflow, resolution curves, particle distribution, map colored by local resolution as determined in Relion. Color scale in Å. Gold-standard FSC plot between two half-maps separately refined in Relion (orange) indicates a resolution of 3.1 Å (0.143 threshold). Map-to-model FSC for the final refined model and the Relion map (blue) indicates a resolution of 3.3 Å (0.5 FSC criterion). **b** Sandwich KtrAB complex: representative micrograph, selected 2D classes, processing workflow, resolution curves, particle distribution, map colored by local resolution as determined in Relion, color scale in Å. Gold-standard FSC (orange curve): 2.82 Å (FSC 0.143), map-to-model FSC (blue): 2.96 Å (FSC 0.5).

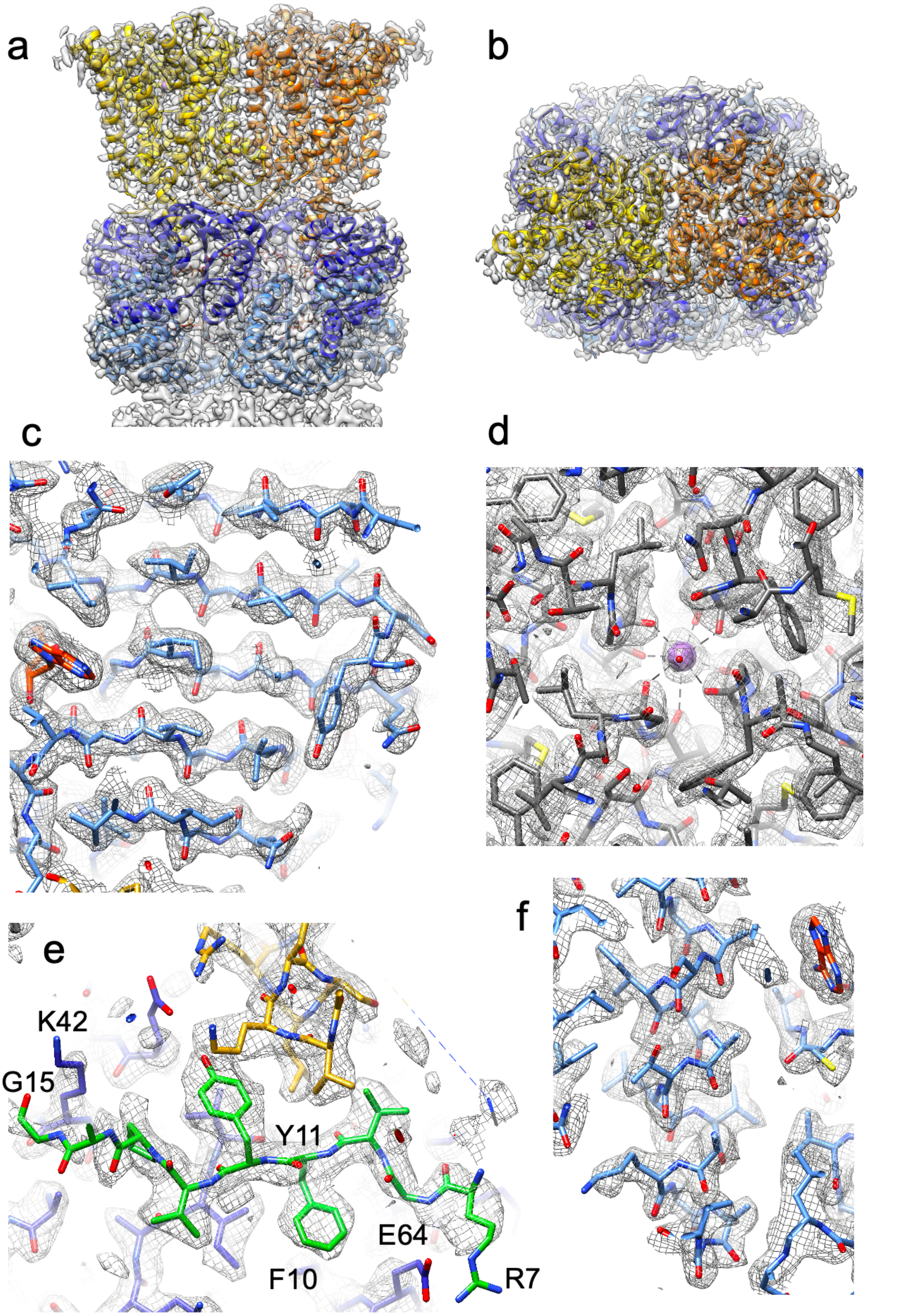

Supplementary Fig. 3: The 2.5 Å cryo-EM map with fitted model. Overview from the side (a) and from the cytoplasm (**b**) KtrA in shades of blue and KtrB in shades of yellow. **c** Beta sheet in KtrA with ADP (orange). **d** Potassium ion (purple) in KtrB as seen from the periplasm coordinated by 8 backbone oxygens. A water molecule (red) sits above it. **e** KtrB N-terminus 7-15 (green) as seen from the periplasm passes the extended D1M2 helix (goldenrod) and lies on top of KtrA (blue). **f** Alpha helix in KtrA.

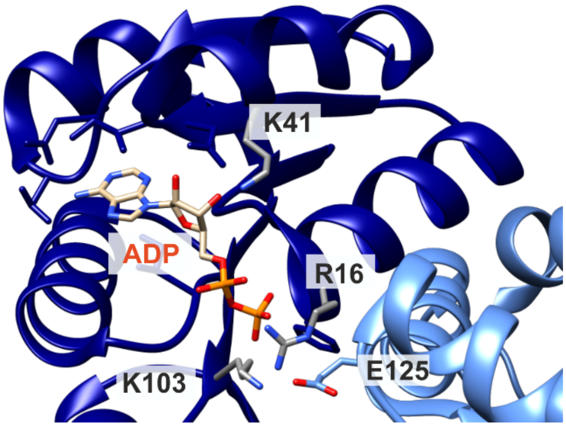

Supplementary Fig. 4: Coordination of ADP by KtrA from *B. subtilis*. ADP-bound crystal structure of KtrA from *B. subtilis* solved at a resolution of 2.93 Å (PDB 4J91). Residues R16 and K103 are involved in coordination of the ADP molecule[17].

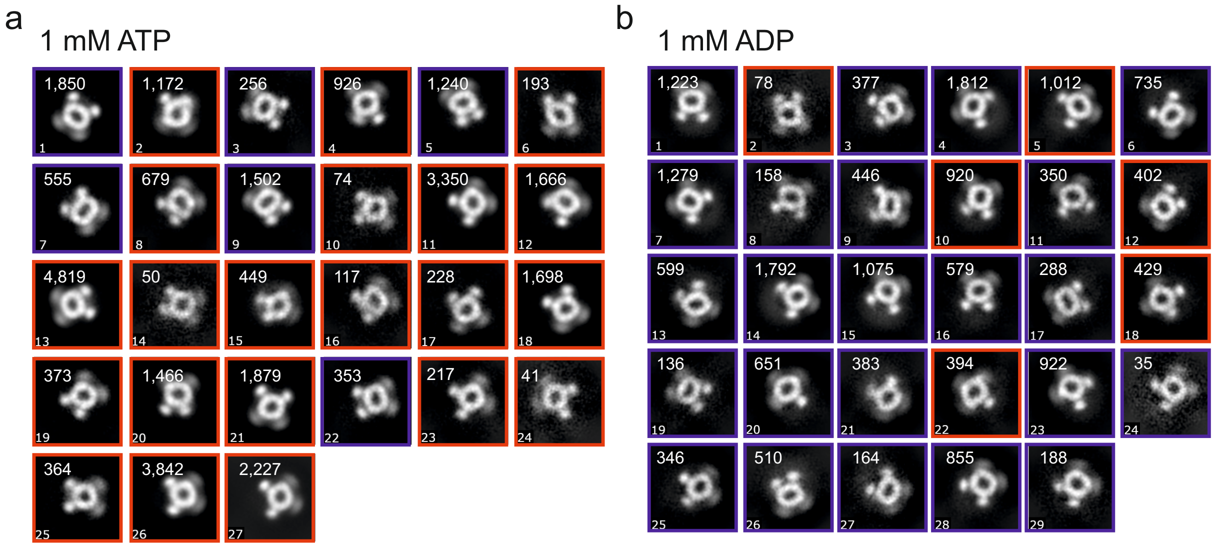

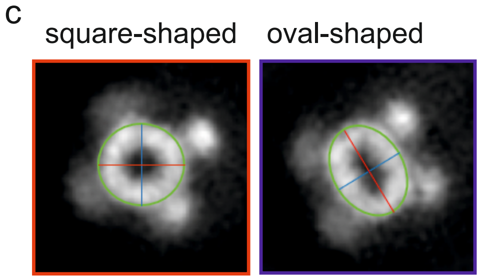

	square	oval
**1 mM ATP**	82%	18%
**1 mM ADP**	18%	82%

Supplementary Fig. 5: 2D class averages of KtrA from *V. alginolyticus* in the presence of nucleotides. KtrA was purified via SEC and the protein at a final concentration of 235 nM was incubated with either 1 mM ADP + 2 mM $MgCl_2$ (**a**), or 1 mM ATP +2 mM $MgCl_2$ (**b**). 3 µl of the sample were applied on a carbon-coated copper grid and stained with 1% uranyl formate. Images were acquired using a Tecnai G2 Spirit Biotwin microscope, a Rio16 camera and Leginon software. Particle picking and 2D classification were performed with cisTEM. The best out of 30 classes are shown. **c** To determine the conformation of the KtrA rings, the ratio between the longest (red line) and the shortest (blue line) axes was determined. A threshold was set to 1.115, and classes with a ratio greater than 1.115 were defined as oval-shaped (blue frame), and classes with a ratio smaller than 1.115 were defined as square-shaped (red frame). This analysis revealed that in the presence ATP the majority (82%) of the particles are in the square-shaped conformation, while in the presence of ADP the majority (82%) adopts an oval shape.

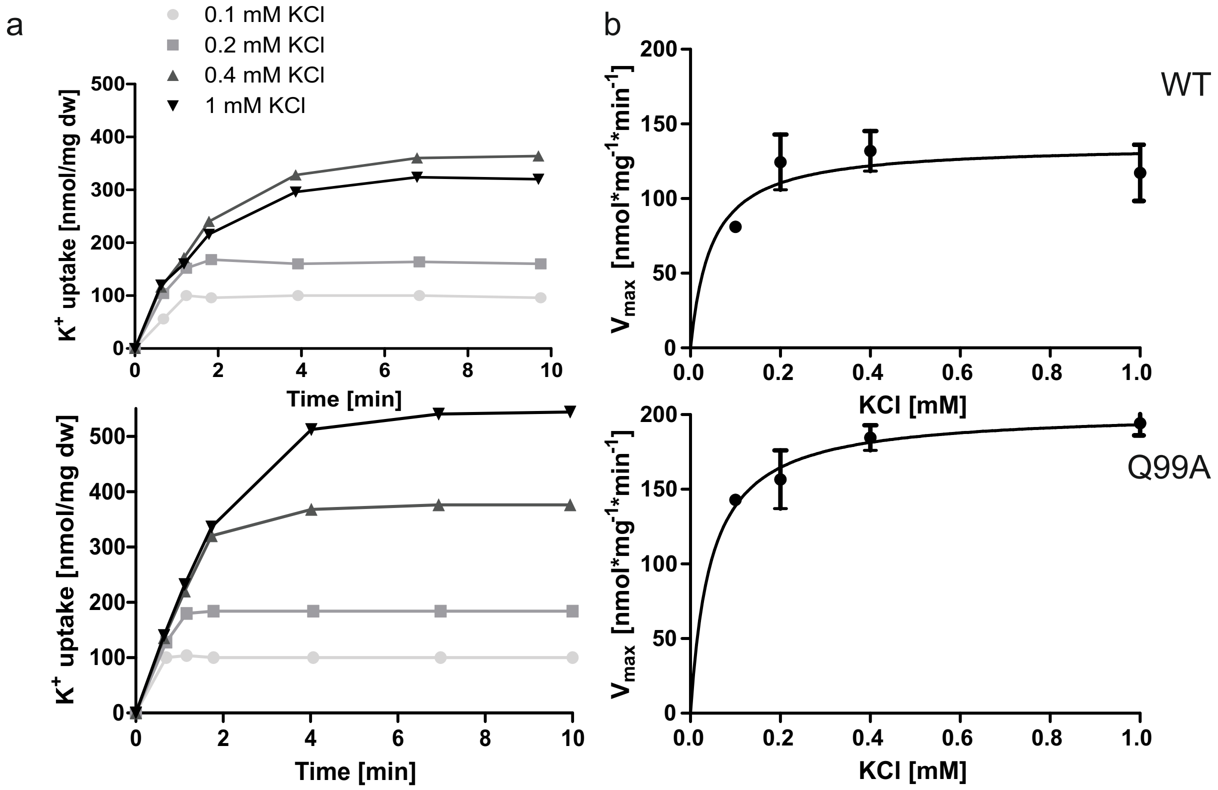

Supplementary Fig. 6: K$^+$ uptake by *E. coli* LB2003 cells producing KtrAB pore variants. **a** After production of KtrAB or KtrAB$_{Q99A}$, *E. coli* LB2003 cells were depleted of K$^+$ and, after 10 min incubation at room temperature, different K$^+$ concentrations (0.1, 0.2, 0.4, and 1 mM, different shades of grey) were added. For 10 min, 1 ml samples were taken at different time points, and cells were separated from medium via centrifugation through silicone oil. Intracellular potassium concentrations were determined by flame photometry. Each set of experiments was performed in biological triplicates, with representative results from a single set shown. **b** Initial uptake velocities are plotted against the KCl concentrations. Error bars represent SDs between the triplicates. Michaelis-Menten fit was performed to determine V_{max} and $K_{m.}$ Average K_m and V_{max} values ± SDs are represented in the main text (Fig. 4f).

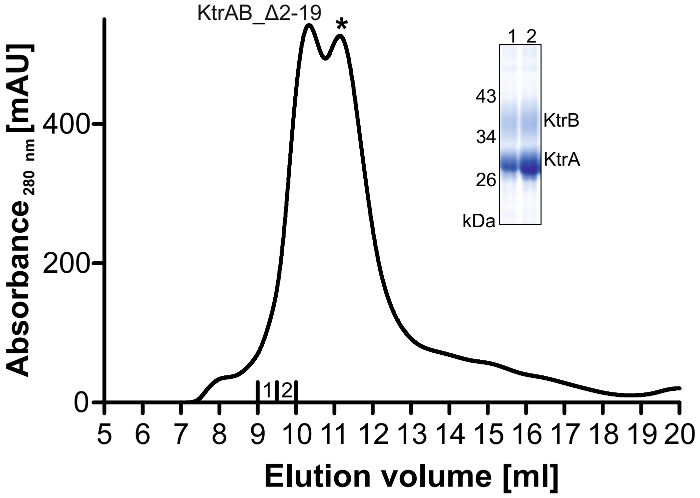

Supplementary Fig. 7: Size-exclusion chromatography of purified $KtrA_8B\Delta2\text{-}19_4$: Elution profile of the KtrAB complex with N-terminus deletion performed on a Superdex® 200 Increase 10/300 GL column. In addition to $KtrA_8B\Delta2\text{-}19_4$, free KtrA rings co-elute at higher elution volumes (indicated by *). Peak fractions 1 and 2 contain both subunits (KtrA: 28 kDa; KtrB: 50kDa, migrates at 37 kDa), indicated by the Coomassie-stained 12% SDS PAGE (inset).

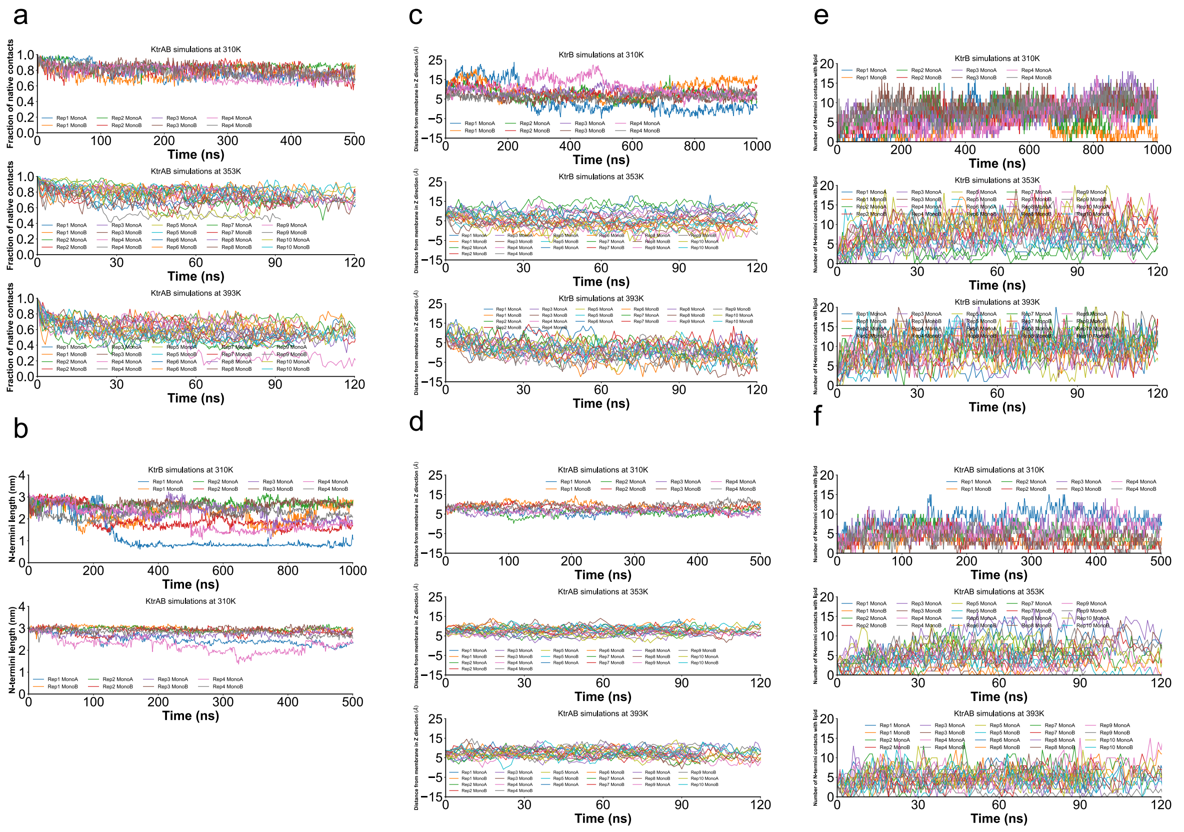

Supplementary Fig. 8: Time series of analysis of all-atom MD simulations of KtrB dimer and the KtrAB complex in a lipid environment. **a** The fraction of native contacts between KtrA and the N-termini of KtrB remain high in the KtrAB complex even at elevated temperatures. **b** The length of KtrB's N-termini varies in the KtrB dimer showing their unstructured nature. In the KtrAB complex, it stays rigid in length as it remains in pocket between the subunits. **c,d** Distributions of the distance of the KtrB N-termini from the membrane surface in the z direction (in Å) in the MD simulations of the KtrB dimer (**c**) and the full KtrAB complex (**d**). **e,f** Distribution of the number of contacts formed between lipids and N-terminal residues of KtrB (**e**) and KtrAB (**f**).

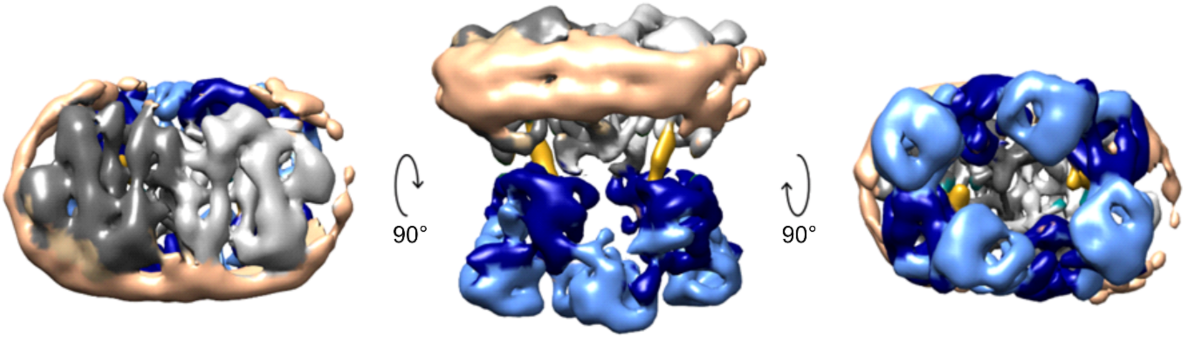

Supplementary Fig. 9: KtrAB reconstituted in MSP2N2 nanodiscs. Purified KtrAB was reconstituted into MSP2N2 in a KtrAB:lipid:MSP ratio of 1:100:2.5. *E. coli* polar lipids were used. A cryo-EM data set yielded a 6 Å resolution map of KtrAB adopting the ADP-bound conformation. The MSP2N2 belt (beige) is tightly wrapped around the transmembrane KtrB subunits (top view – left panel, bottom view – right panel).

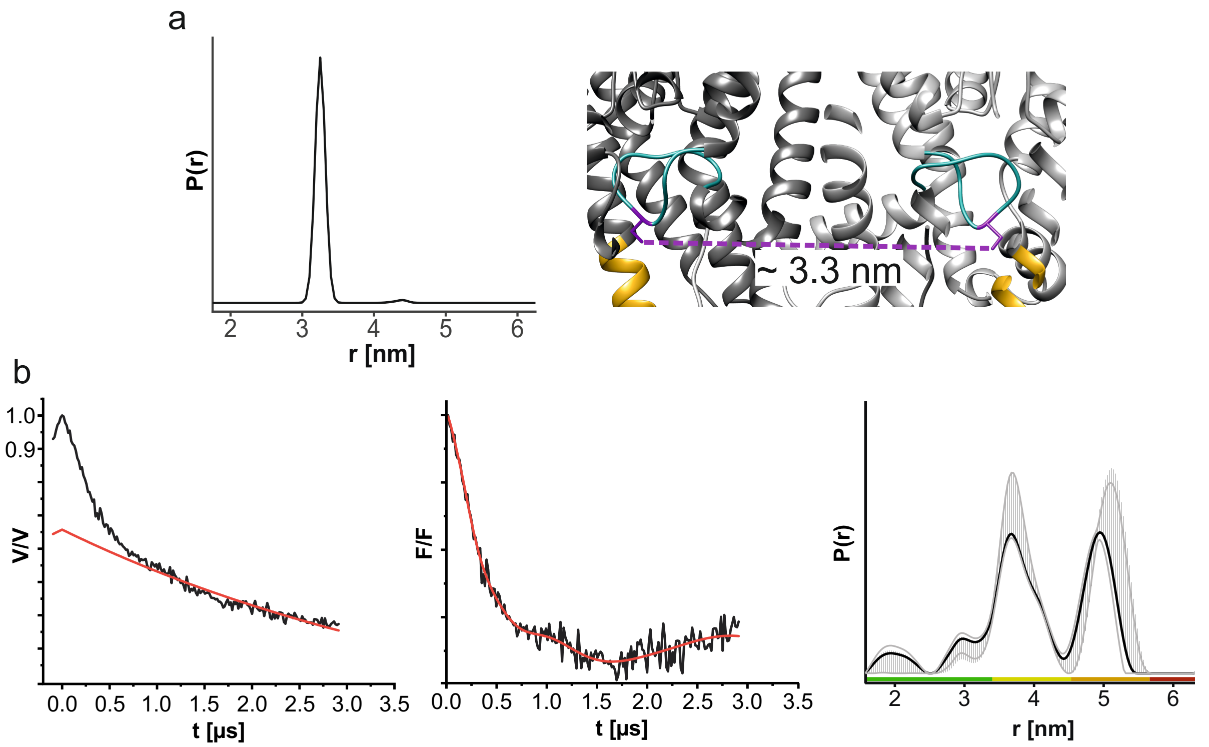

Supplementary Fig. 10: Flexibility of the intramembrane loop of KtrB in the absence of KtrA. a Predicted distance distribution of spin-labelled residues 318C in ADP-bound KtrAB (7zp9) resulting from a rotamer library analysis using MMM2020.1 **b** DEER measurements of $KtrB_{T318C\_R1}$ (R1 indicates spin labelling) variant reconstituted in liposomes. Left panel: Experimental raw data V(t) with fitted background function; middle panel: Background-corrected dipolar evolution function F(t); right panel: Interspin distance distribution P(r) obtained by Tikhonov regularization. Grey areas represent the full variation of possible distance distributions. The lower and upper error estimates (grey lines) represent the respective mean values minus and plus twice the standard deviation. Distance is measured between the labelled residues in the two KtrB protomers.

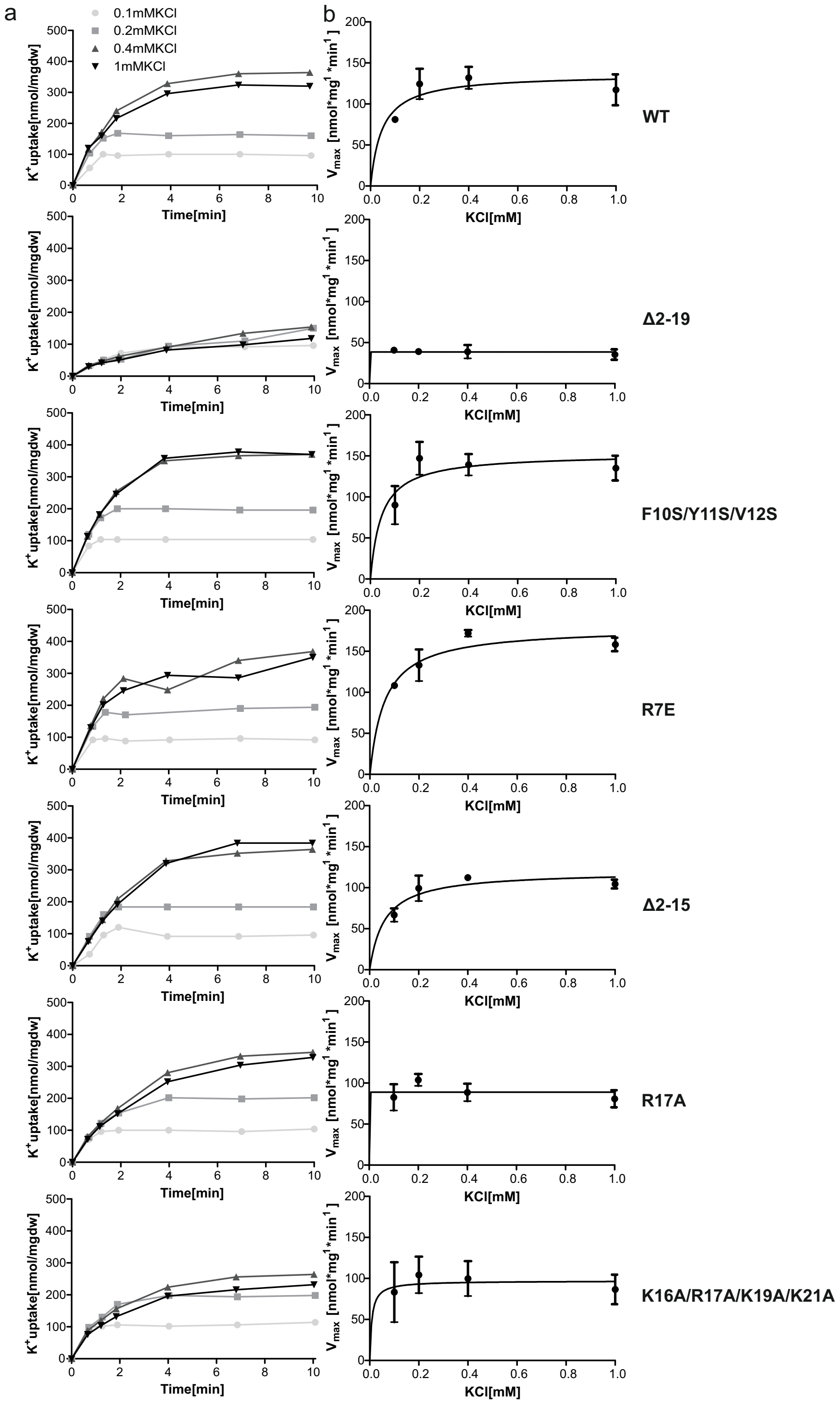

Supplementary Fig. 11: Uptake by *E. coli* LB2003 cells producing KtrAB with variations in the N-terminus of KtrB. a After production of KtrAB with different variations in the N-terminus, *E. coli* LB2003 cells were depleted of K^+ and, after 10 min incubation at room temperature, different K^+ concentrations (0.1, 0.2, 0.4, and 1 mM, different shades of black) were added. For 10 min, 1 ml samples were taken at different time points and cells were separated from medium via centrifugation through silicone oil. Intracellular potassium concentrations were determined by flame photometry. Each set of experiments was performed in biological triplicates, with representative results from a single set shown. **b** Initial uptake velocities are plotted against the KCl concentrations. Error bars represent SDs from triplicates. Michaelis-Menten fit was performed to determine V_{max} and K_{m}. Average K_m and V_{max} values ± SDs are represented in the main text (Fig. 9 a, b).

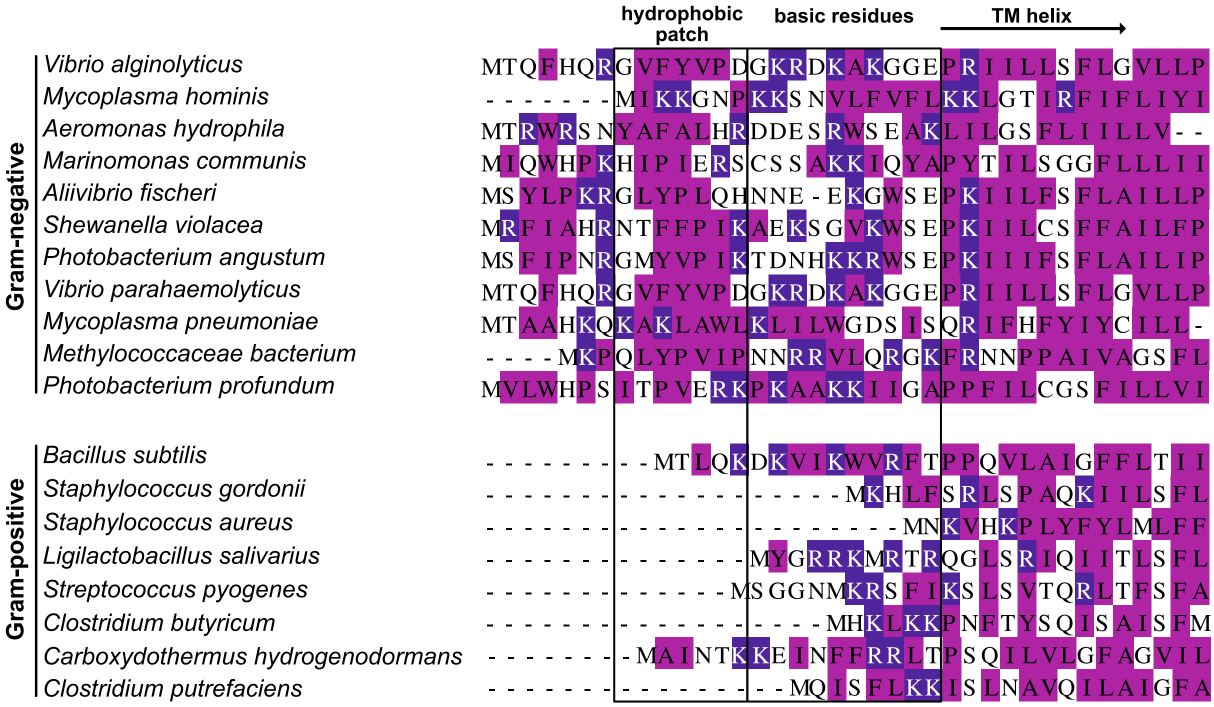

Supplementary Fig. 12: Sequence alignment of N-termini from KtrB of different bacterial species. The N-termini were defined as the peptides from the first amino acid of the proteins to the first transmembrane helix (predicted by PROMALS3D server[40]). Multiple sequence alignment was performed using Clustal Omega server and edited using JalView[41]. Hydrophobic residues and basic residues are labelled pink and blue, respectively. The hydrophobic patch and the cluster of basic residues are highlighted by black boxes.

Supplementary Table 1: Cryo-EM data collection, refinement and model statistics

	$KtrB_2A_8B_2$	Native complex $KtrB_2A_8$	$KtrB_2A_8B_2$ ΔKtrB1-19
**Accession codes**	EMD-14851<br>PDB 7zp9	EMD-14859<br>PDB 7zpo	EMD-14862<br>PDB 7zpr
**Data collection**			
Microscope	Titan Krios G2	Titan Krios G2	Titan Krios G2
Camera	Gatan K3 Summit	Gatan K3 Summit	Gatan K3 Summit
Voltage (kV)	300	300	300
Nominal magnification	105,000x	105,000x	105,000x
Calibrated pixel size (Å)	0.831	0.831	0.831
Electron exposure ($e^-/Å^2$)	40.0	40.0	40.0
Exposure time (s)	2	2	2
Number of frames per image	40	40	40
Defocus range (µm)	-1.0 – -2.5	-0.8 – -2.5	-0.5 – -2.2
**Image processing**			
Motion correction software	*MotionCor2*	*MotionCor2*	*MotionCor2*
CTF estimation software	*Gctf*	*Gctf*	*Gctf*
Particle selection software	*crYOLO*	*crYOLO*	*crYOLO*
Micrographs (no.)	5,956	3,663	7,109
Initial particle images (no.)	198,298	394,476	211,579
Final particle images (no.)	73,965	211,602	58,420
Symmetry	D2	C2	D2
Applied *B*-factor ($Å^2$)	-65	-100	-160
Final resolution (Relion) (Å)	2.8	3.1	3.6
Final resolution (denmod) (Å)	2.5	2.8	ND
**Refinement statistics**			
Modeling software	*COOT, PHENIX*	*COOT, PHENIX*	*COOT, PHENIX*
Protein residues	2802	1932	2766
Ligands	8 ADP, 8 Mg,<br>4 K, 36 LMT	8 ADP, 8 Mg,<br>2 K, 4 LMT	8 ADP, 8 Mg,<br>4 K
RMS deviations			
Bond lengths (Å)	0.33	0.25	0.33
Bond angles (°)	0.48	0.46	0.48
Ramachandran plot			
Outliers (%)	0.05	0.00	0.22
Allowed (%)	2.26	1.31	1.83
Favored (%)	97.69	98.69	97.95
Rotamer outliers (%)	0.64	0.00	0.89
Molprobity score	1.62	1.55	1.60
All-atom clash score	10.77	10.71	9.45

Supplementary Table 2: Description of the molecular dynamics simulations performed with corresponding temperature and length.

Simulation target	Temperature	Total length of simulation
KtrB	310 K	4 μs (4 $\times$ 1 μs)
KtrB	353 K	1.6 μs (5 $\times$ 200 ns & 5 $\times$ 120 ns)
KtrB	393 K	1.6 μs (5 $\times$ 200 ns & 5 $\times$ 120 ns)
KtrAB	310 K	2.5 μs (1 $\times$ 1 μs & 3 $\times$ 0.5 μs)
KtrAB	353 K	1.6 μs (5 $\times$ 200 ns & 5 $\times$ 120 ns)
KtrAB	353 K	1.6 μs (5 $\times$ 200 ns & 5 $\times$ 120 ns)
