# Supplementary material for: A short intrinsically disordered region at KtrB’s N-terminus facilitates allosteric regulation of K+ channel KtrAB
Source: Nat Commun. 2025 May 7;16:4252. doi: 10.1038/s41467-025-59546-z (PMC12059179; doi:10.1038/s41467-025-59546-z)
Supplement: Supplementary file 2 — Description of Additional Supplementary Files [file 41467_2025_59546_MOESM2_ESM.pdf]

Description of Additional Supplementary Files

File Name: Supplementary Movie 1

Description: Intrinsically disordered KtrB N-terminus interacts with the membrane. Representative MD simulations containing dimeric KtrB in a symmetric heterogeneous bilayer (POPE/POPG/CL). Individual lipids are represented in grey licorice. The KtrB dimer is represented in cyan ribbon, and the interacting N-terminus residues are shown in sticks. The movie spans 1 μs from one of the replicates.
